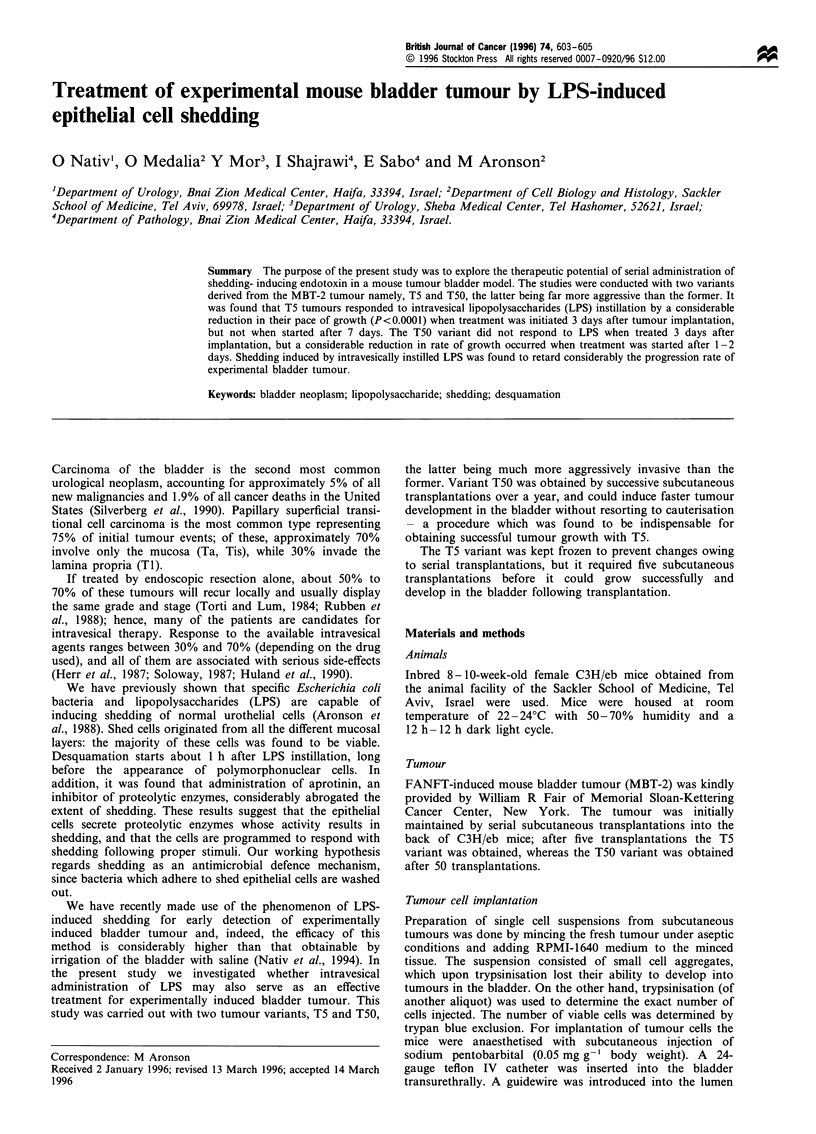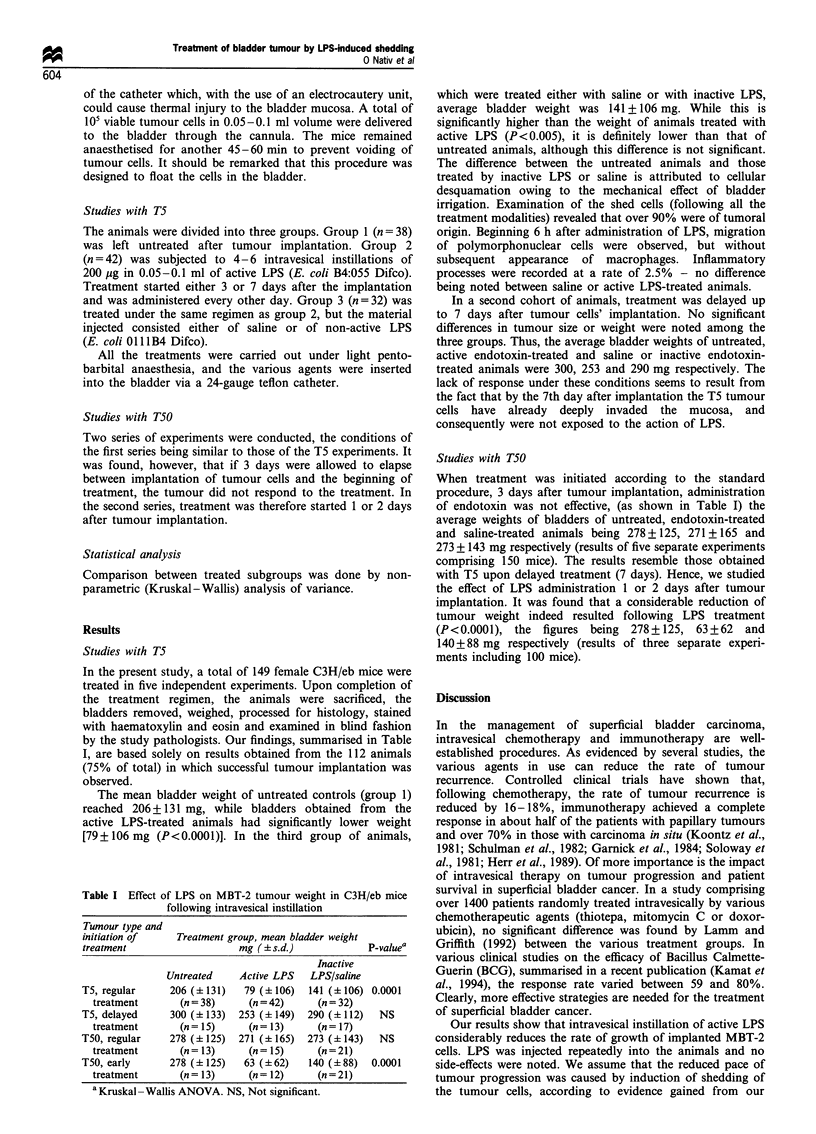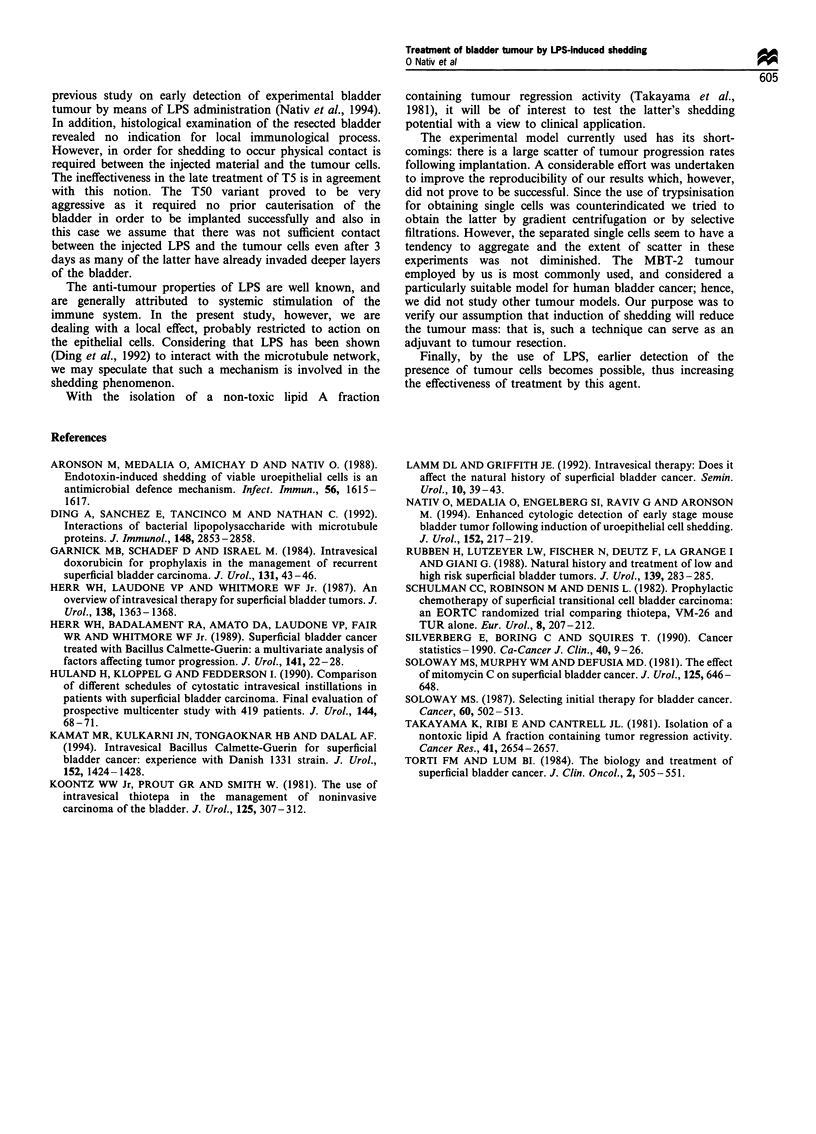# Treatment of experimental mouse bladder tumour by LPS-induced epithelial cell shedding.

**DOI:** 10.1038/bjc.1996.408

**Published:** 1996-08

**Authors:** O. Nativ, O. Medalia, Y. Mor, I. Shajrawi, E. Sabo, M. Aronson

**Affiliations:** Department of Urology, Bnai Zion Medical Center, Haifa, Israel.

## Abstract

The purpose of the present study was to explore the therapeutic potential of serial administration of shedding-inducing endotoxin in a mouse tumour bladder model. The studies were conducted with two variants derived from the MBT-2 tumour namely, T5 and T50, the latter being far more aggressive than the former. It was found that T5 tumours responded to intravesical lipopolysaccharides (LPS) instillation by a considerable reduction in their pace of growth (P < 0.0001) when treatment was initiated 3 days after tumour implantation, but not when started after 7 days. The T50 variant did not respond to LPS when treated 3 days after implantation, but a considerable reduction in rate of growth occurred when treatment was started after 1-2 days. Shedding induced by intravesically instilled LPS was found to retard considerably the progression rate of experimental bladder tumour.


British Journal of Cancer (1996) 74, 603-605
© 1996 Stockton Press All rights reserved 0007-0920/96 $12.00

### Treatment of experimental mouse bladder tumour by LPS-induced epithelial cell shedding

O Nativ[1], O Medalia[2] Y Mor[3], I Shajrawi[4], E Sabo[4] and M Aronson[2]

[1]*Department of Urology, Bnai Zion Medical Center, Haifa, 33394, Israel;* [2]*Department of Cell Biology and Histology, Sackler School of Medicine, Tel Aviv, 69978, Israel;* [3]*Department of Urology, Sheba Medical Center, Tel Hashomer, 52621, Israel;* [4]*Department of Pathology, Bnai Zion Medical Center, Haifa, 33394, Israel.*

**Summary** The purpose of the present study was to explore the therapeutic potential of serial administration of shedding- inducing endotoxin in a mouse tumour bladder model. The studies were conducted with two variants derived from the MBT-2 tumour namely, T5 and T50, the latter being far more aggressive than the former. It was found that T5 tumours responded to intravesical lipopolysaccharides (LPS) instillation by a considerable reduction in their pace of growth ($P < 0.0001$) when treatment was initiated 3 days after tumour implantation, but not when started after 7 days. The T50 variant did not respond to LPS when treated 3 days after implantation, but a considerable reduction in rate of growth occurred when treatment was started after 1-2 days. Shedding induced by intravesically instilled LPS was found to retard considerably the progression rate of experimental bladder tumour.

**Keywords:** bladder neoplasm; lipopolysaccharide; shedding; desquamation

Carcinoma of the bladder is the second most common urological neoplasm, accounting for approximately 5% of all new malignancies and 1.9% of all cancer deaths in the United States (Silverberg *et al.*, 1990). Papillary superficial transitional cell carcinoma is the most common type representing 75% of initial tumour events; of these, approximately 70% involve only the mucosa (Ta, Tis), while 30% invade the lamina propria (T1).

If treated by endoscopic resection alone, about 50% to 70% of these tumours will recur locally and usually display the same grade and stage (Torti and Lum, 1984; Rubben *et al.*, 1988); hence, many of the patients are candidates for intravesical therapy. Response to the available intravesical agents ranges between 30% and 70% (depending on the drug used), and all of them are associated with serious side-effects (Herr *et al.*, 1987; Soloway, 1987; Huland *et al.*, 1990).

We have previously shown that specific *Escherichia coli* bacteria and lipopolysaccharides (LPS) are capable of inducing shedding of normal urothelial cells (Aronson *et al.*, 1988). Shed cells originated from all the different mucosal layers: the majority of these cells was found to be viable. Desquamation starts about 1 h after LPS instillation, long before the appearance of polymorphonuclear cells. In addition, it was found that administration of aprotinin, an inhibitor of proteolytic enzymes, considerably abrogated the extent of shedding. These results suggest that the epithelial cells secrete proteolytic enzymes whose activity results in shedding, and that the cells are programmed to respond with shedding following proper stimuli. Our working hypothesis regards shedding as an antimicrobial defence mechanism, since bacteria which adhere to shed epithelial cells are washed out.

We have recently made use of the phenomenon of LPS-induced shedding for early detection of experimentally induced bladder tumour and, indeed, the efficacy of this method is considerably higher than that obtainable by irrigation of the bladder with saline (Nativ *et al.*, 1994). In the present study we investigated whether intravesical administration of LPS may also serve as an effective treatment for experimentally induced bladder tumour. This study was carried out with two tumour variants, T5 and T50, the latter being much more aggressively invasive than the former. Variant T50 was obtained by successive subcutaneous transplantations over a year, and could induce faster tumour development in the bladder without resorting to cauterisation – a procedure which was found to be indispensable for obtaining successful tumour growth with T5.

The T5 variant was kept frozen to prevent changes owing to serial transplantations, but it required five subcutaneous transplantations before it could grow successfully and develop in the bladder following transplantation.

### Materials and methods

### *Animals*

Inbred 8-10-week-old female C3H/eb mice obtained from the animal facility of the Sackler School of Medicine, Tel Aviv, Israel were used. Mice were housed at room temperature of 22-24°C with 50-70% humidity and a 12 h-12 h dark light cycle.

### *Tumour*

FANFT-induced mouse bladder tumour (MBT-2) was kindly provided by William R Fair of Memorial Sloan-Kettering Cancer Center, New York. The tumour was initially maintained by serial subcutaneous transplantations into the back of C3H/eb mice; after five transplantations the T5 variant was obtained, whereas the T50 variant was obtained after 50 transplantations.

### *Tumour cell implantation*

Preparation of single cell suspensions from subcutaneous tumours was done by mincing the fresh tumour under aseptic conditions and adding RPMI-1640 medium to the minced tissue. The suspension consisted of small cell aggregates, which upon trypsinisation lost their ability to develop into tumours in the bladder. On the other hand, trypsinisation (of another aliquot) was used to determine the exact number of cells injected. The number of viable cells was determined by trypan blue exclusion. For implantation of tumour cells the mice were anaesthetised with subcutaneous injection of sodium pentobarbital (0.05 mg $g^{-1}$ body weight). A 24-gauge teflon IV catheter was inserted into the bladder transurethrally. A guidewire was introduced into the lumen

Correspondence: M Aronson
Received 2 January 1996; revised 13 March 1996; accepted 14 March 1996

of the catheter which, with the use of an electrocautery unit, could cause thermal injury to the bladder mucosa. A total of $10^5$ viable tumour cells in 0.05–0.1 ml volume were delivered to the bladder through the cannula. The mice remained anaesthetised for another 45–60 min to prevent voiding of tumour cells. It should be remarked that this procedure was designed to float the cells in the bladder.

*Studies with T5*

The animals were divided into three groups. Group 1 ($n=38$) was left untreated after tumour implantation. Group 2 ($n=42$) was subjected to 4–6 intravesical instillations of 200 $\mu$g in 0.05–0.1 ml of active LPS (*E. coli* B4:055 Difco). Treatment started either 3 or 7 days after the implantation and was administered every other day. Group 3 ($n=32$) was treated under the same regimen as group 2, but the material injected consisted either of saline or of non-active LPS (*E. coli* 0111B4 Difco).

All the treatments were carried out under light pentobarbital anaesthesia, and the various agents were inserted into the bladder via a 24-gauge teflon catheter.

*Studies with T50*

Two series of experiments were conducted, the conditions of the first series being similar to those of the T5 experiments. It was found, however, that if 3 days were allowed to elapse between implantation of tumour cells and the beginning of treatment, the tumour did not respond to the treatment. In the second series, treatment was therefore started 1 or 2 days after tumour implantation.

*Statistical analysis*

Comparison between treated subgroups was done by non-parametric (Kruskal–Wallis) analysis of variance.

### Results

*Studies with T5*

In the present study, a total of 149 female C3H/eb mice were treated in five independent experiments. Upon completion of the treatment regimen, the animals were sacrificed, the bladders removed, weighed, processed for histology, stained with haematoxylin and eosin and examined in blind fashion by the study pathologists. Our findings, summarised in Table I, are based solely on results obtained from the 112 animals (75% of total) in which successful tumour implantation was observed.

The mean bladder weight of untreated controls (group 1) reached $206 \pm 131$ mg, while bladders obtained from the active LPS-treated animals had significantly lower weight [$79 \pm 106$ mg ($P<0.0001$)]. In the third group of animals, which were treated either with saline or with inactive LPS, average bladder weight was $141 \pm 106$ mg. While this is significantly higher than the weight of animals treated with active LPS ($P<0.005$), it is definitely lower than that of untreated animals, although this difference is not significant. The difference between the untreated animals and those treated by inactive LPS or saline is attributed to cellular desquamation owing to the mechanical effect of bladder irrigation. Examination of the shed cells (following all the treatment modalities) revealed that over 90% were of tumoral origin. Beginning 6 h after administration of LPS, migration of polymorphonuclear cells were observed, but without subsequent appearance of macrophages. Inflammatory processes were recorded at a rate of 2.5% – no difference being noted between saline or active LPS-treated animals.

In a second cohort of animals, treatment was delayed up to 7 days after tumour cells' implantation. No significant differences in tumour size or weight were noted among the three groups. Thus, the average bladder weights of untreated, active endotoxin-treated and saline or inactive endotoxin-treated animals were 300, 253 and 290 mg respectively. The lack of response under these conditions seems to result from the fact that by the 7th day after implantation the T5 tumour cells have already deeply invaded the mucosa, and consequently were not exposed to the action of LPS.

*Studies with T50*

When treatment was initiated according to the standard procedure, 3 days after tumour implantation, administration of endotoxin was not effective, (as shown in Table I) the average weights of bladders of untreated, endotoxin-treated and saline-treated animals being $278 \pm 125$, $271 \pm 165$ and $273 \pm 143$ mg respectively (results of five separate experiments comprising 150 mice). The results resemble those obtained with T5 upon delayed treatment (7 days). Hence, we studied the effect of LPS administration 1 or 2 days after tumour implantation. It was found that a considerable reduction of tumour weight indeed resulted following LPS treatment ($P<0.0001$), the figures being $278 \pm 125$, $63 \pm 62$ and $140 \pm 88$ mg respectively (results of three separate experiments including 100 mice).

**Table I** Effect of LPS on MBT-2 tumour weight in C3H/eb mice following intravesical instillation

| *Tumour type and initiation of treatment* | *Treatment group, mean bladder weight mg (±s.d.)* | | | P-*value*[a] |
|---|---|---|---|---|
| | *Untreated* | *Active LPS* | *Inactive LPS/saline* | |
| T5, regular treatment | 206 (±131) ($n=38$) | 79 (±106) ($n=42$) | 141 (±106) ($n=32$) | 0.0001 |
| T5, delayed treatment | 300 (±133) ($n=15$) | 253 (±149) ($n=13$) | 290 (±112) ($n=17$) | NS |
| T50, regular treatment | 278 (±125) ($n=13$) | 271 (±165) ($n=15$) | 273 (±143) ($n=21$) | NS |
| T50, early treatment | 278 (±125) ($n=13$) | 63 (±62) ($n=12$) | 140 (±88) ($n=21$) | 0.0001 |

[a] Kruskal–Wallis ANOVA. NS, Not significant.

### Discussion

In the management of superficial bladder carcinoma, intravesical chemotherapy and immunotherapy are well-established procedures. As evidenced by several studies, the various agents in use can reduce the rate of tumour recurrence. Controlled clinical trials have shown that, following chemotherapy, the rate of tumour recurrence is reduced by 16–18%, immunotherapy achieved a complete response in about half of the patients with papillary tumours and over 70% in those with carcinoma *in situ* (Koontz *et al.*, 1981; Schulman *et al.*, 1982; Garnick *et al.*, 1984; Soloway *et al.*, 1981; Herr *et al.*, 1989). Of more importance is the impact of intravesical therapy on tumour progression and patient survival in superficial bladder cancer. In a study comprising over 1400 patients randomly treated intravesically by various chemotherapeutic agents (thiotepa, mitomycin C or doxorubicin), no significant difference was found by Lamm and Griffith (1992) between the various treatment groups. In various clinical studies on the efficacy of Bacillus Calmette-Guerin (BCG), summarised in a recent publication (Kamat *et al.*, 1994), the response rate varied between 59 and 80%. Clearly, more effective strategies are needed for the treatment of superficial bladder cancer.

Our results show that intravesical instillation of active LPS considerably reduces the rate of growth of implanted MBT-2 cells. LPS was injected repeatedly into the animals and no side-effects were noted. We assume that the reduced pace of tumour progression was caused by induction of shedding of the tumour cells, according to evidence gained from our previous study on early detection of experimental bladder tumour by means of LPS administration (Nativ *et al.*, 1994). In addition, histological examination of the resected bladder revealed no indication for local immunological process. However, in order for shedding to occur physical contact is required between the injected material and the tumour cells. The ineffectiveness in the late treatment of T5 is in agreement with this notion. The T50 variant proved to be very aggressive as it required no prior cauterisation of the bladder in order to be implanted successfully and also in this case we assume that there was not sufficient contact between the injected LPS and the tumour cells even after 3 days as many of the latter have already invaded deeper layers of the bladder.

The anti-tumour properties of LPS are well known, and are generally attributed to systemic stimulation of the immune system. In the present study, however, we are dealing with a local effect, probably restricted to action on the epithelial cells. Considering that LPS has been shown (Ding *et al.*, 1992) to interact with the microtubule network, we may speculate that such a mechanism is involved in the shedding phenomenon.

With the isolation of a non-toxic lipid A fraction containing tumour regression activity (Takayama *et al.*, 1981), it will be of interest to test the latter's shedding potential with a view to clinical application.

The experimental model currently used has its shortcomings: there is a large scatter of tumour progression rates following implantation. A considerable effort was undertaken to improve the reproducibility of our results which, however, did not prove to be successful. Since the use of trypsinisation for obtaining single cells was counterindicated we tried to obtain the latter by gradient centrifugation or by selective filtrations. However, the separated single cells seem to have a tendency to aggregate and the extent of scatter in these experiments was not diminished. The MBT-2 tumour employed by us is most commonly used, and considered a particularly suitable model for human bladder cancer; hence, we did not study other tumour models. Our purpose was to verify our assumption that induction of shedding will reduce the tumour mass: that is, such a technique can serve as an adjuvant to tumour resection.

Finally, by the use of LPS, earlier detection of the presence of tumour cells becomes possible, thus increasing the effectiveness of treatment by this agent.

### References

ARONSON M, MEDALIA O, AMICHAY D AND NATIV O. (1988). Endotoxin-induced shedding of viable uroepithelial cells is an antimicrobial defence mechanism. *Infect. Immun.*, **56**, 1615–1617.

DING A, SANCHEZ E, TANCINCO M AND NATHAN C. (1992). Interactions of bacterial lipopolysaccharide with microtubule proteins. *J. Immunol.*, **148**, 2853–2858.

GARNICK MB, SCHADEF D AND ISRAEL M. (1984). Intravesical doxorubicin for prophylaxis in the management of recurrent superficial bladder carcinoma. *J. Urol.*, **131**, 43–46.

HERR WH, LAUDONE VP AND WHITMORE WF Jr. (1987). An overview of intravesical therapy for superficial bladder tumors. *J. Urol.*, **138**, 1363–1368.

HERR WH, BADALAMENT RA, AMATO DA, LAUDONE VP, FAIR WR AND WHITMORE WF Jr. (1989). Superficial bladder cancer treated with Bacillus Calmette-Guerin: a multivariate analysis of factors affecting tumor progression. *J. Urol.*, **141**, 22–28.

HULAND H, KLOPPEL G AND FEDDERSON I. (1990). Comparison of different schedules of cytostatic intravesical instillations in patients with superficial bladder carcinoma. Final evaluation of prospective multicenter study with 419 patients. *J. Urol.*, **144**, 68–71.

KAMAT MR, KULKARNI JN, TONGAOKNAR HB AND DALAL AF. (1994). Intravesical Bacillus Calmette-Guerin for superficial bladder cancer: experience with Danish 1331 strain. *J. Urol.*, **152**, 1424–1428.

KOONTZ WW Jr, PROUT GR AND SMITH W. (1981). The use of intravesical thiotepa in the management of noninvasive carcinoma of the bladder. *J. Urol.*, **125**, 307–312.

LAMM DL AND GRIFFITH JE. (1992). Intravesical therapy: Does it affect the natural history of superficial bladder cancer. *Semin. Urol.*, **10**, 39–43.

NATIV O, MEDALIA O, ENGELBERG SI, RAVIV G AND ARONSON M. (1994). Enhanced cytologic detection of early stage mouse bladder tumor following induction of uroepithelial cell shedding. *J. Urol.*, **152**, 217–219.

RUBBEN H, LUTZEYER LW, FISCHER N, DEUTZ F, LA GRANGE I AND GIANI G. (1988). Natural history and treatment of low and high risk superficial bladder tumors. *J. Urol.*, **139**, 283–285.

SCHULMAN CC, ROBINSON M AND DENIS L. (1982). Prophylactic chemotherapy of superficial transitional cell bladder carcinoma: an EORTC randomized trial comparing thiotepa, VM-26 and TUR alone. *Eur. Urol.*, **8**, 207–212.

SILVERBERG E, BORING C AND SQUIRES T. (1990). Cancer statistics – 1990. *Ca-Cancer J. Clin.*, **40**, 9–26.

SOLOWAY MS, MURPHY WM AND DEFUSIA MD. (1981). The effect of mitomycin C on superficial bladder cancer. *J. Urol.*, **125**, 646–648.

SOLOWAY MS. (1987). Selecting initial therapy for bladder cancer. *Cancer*, **60**, 502–513.

TAKAYAMA K, RIBI E AND CANTRELL JL. (1981). Isolation of a nontoxic lipid A fraction containing tumor regression activity. *Cancer Res.*, **41**, 2654–2657.

TORTI FM AND LUM BI. (1984). The biology and treatment of superficial bladder cancer. *J. Clin. Oncol.*, **2**, 505–551.